# Dog Tethering in Slovakia: Legal, Ethical and Behavioral Aspects and Dog Welfare Implications

**DOI:** 10.3390/ani11030594

**Published:** 2021-02-24

**Authors:** Daniela Takáčová, Lenka Skurková, Lýdia Mesarčová, Lenka Lešková, Lucia Kottferová, Anna Packová, Dávid Vajányi, Jana Kottferová

**Affiliations:** 1 Workplace of Forensic and Public Veterinary Medicine and Economy, Department of Public Veterinary Medicine and Animal Welfare, University of Veterinary Medicine and Pharmacy, Komenského 73, 04181 Košice, Slovakia; daniela.takacova@uvlf.sk (D.T.); eny.packova@gmail.com (A.P.); 2 Workplace of Applied Ethology and Professional Ethics, Department of Public Veterinary Medicine and Animal Welfare, University of Veterinary Medicine and Pharmacy, Komenského 73, 04181 Košice, Slovakia; lenka.skurkova@uvlf.sk (L.S.); lydia.mesarcova@uvlf.sk (L.M.); lenka.leskova@uvlf.sk (L.L.); david.vajanyi@gmail.com (D.V.); jana.kottferova@uvlf.sk (J.K.); 3 Clinic of Birds, Exotic and Free Living Animals, University Veterinary Hospital, University of Veterinary Medicine and Pharmacy, Komenského 73, 04181 Košice, Slovakia

**Keywords:** animal cruelty, animal welfare, dog chaining, dog tethering, legislation, questionnaire

## Abstract

**Simple Summary:**

On the basis of legislative provisions and the behavioral needs of dogs, the authors describe the potential consequences and negative impacts of the long-term tethering of dogs. Dogs should be kept under conditions that allow them, with respect to their size, temperament, stage of their development, and degree of adaptation, to maintain good health and meet their physiological, ethological, and social needs. Despite the adoption of new legislative provisions, this issue has not yet been resolved and we are still coming across various cases involving cruelty to animals of various character and intensity. The current situation can be changed by adopting legislative regulations that explicitly define the responsibilities of owners/keepers including a ban on the tethering of dogs in order to prevent potential circumvention of the legislative rules.

**Abstract:**

Long-term tethering of dogs, or their keeping under unsuitable conditions can result in issues related to changes in their behavior as they may not satisfy their basic needs of life. These needs are discussed in this paper, along with cases when dogs unnecessarily have to endure cruelty and pain. The unavoidable tethering of a dog must not cause trauma and must be arranged in a way that it guarantees physical comfort. Failure to meet the basic needs of an animal may result in manifestation of fear and subsequent aggressiveness. Owners of animals are responsible for their life and health, and their obligations include eliminating the possibility of them hurting themselves or other beings. The relevant adopted legislative provisions should provide protection to animals and be enforceable, which currently appears rather difficult. Controlling and observation of the legislative provisions related to the tethering of dogs raises some difficulties for animal protection inspectors. It is necessary to focus on the specificities of keeping conditions of various dog breeds and on their individual features. Based on research and the relevant Slovak legislative provisions, this paper discusses various views on the practice of tethering dogs from the point of view of public safety and the ethical consequences of permanent dog tethering. Data on dog tethering in Slovakia were evaluated based on a survey and Slovak legal rules governing this issue were analyzed along with various views of public safety and the ethical consequences of permanent dog tethering.

## 1. Introduction

The majority of dog owners are concerned with regard to suitable housing for their dogs, giving them sufficient exercise, play, and adequate socialization. However, for various reasons, some owners decide to secure their dogs by tethering, either temporarily or permanently.

Tethering of companion dogs is used to limit their movement to prevent their straying, digging under fences, escape, and damage to the property in places that are insufficiently secured, or in cases where household members are afraid of the dog. Chaining of dogs by some owners may be a result of inherited behavior from previous generations, whilst other owners use it as a form of cruelty to dogs, either unaware or deliberate. Other examples are sled dogs or dogs involved in some sports or research where these animals are tethered to ensure more accurate observations and physiological measurements [1].

Tethering is the practice of chaining, tying, fastening, or restraining a dog to a ground stake or a stationary object (such as a tree, fence, car, or dog house), usually in a pet owner’s yard, as a means of keeping the dog under control. The term does not refer to a dog being walked on a leash [2]. In similarity with the Victorian Code of Practice [3], these terms are not intended to refer to short-term tying up or with hobbling. Tethering is regarded as a temporary method of restraint that is not suitable for long-term confinement.

According to the Humane Society of the United States [4] the terms “chaining” and “tethering” refer to the practice of fastening a dog to a stationary object while leaving them unattended. The society also defines the difference between these two terms. The term “chaining” tends to refer to situations where thick, heavy chains are used. “Tethering” is more often referred to as partial restraint on a rope, lighter chain, or pulley, which is the more prevalent form of tethering. Risks to public safety and inhumane treatment of dogs are generally considered to be the two primary problems stemming from the permanent tethering of dogs. The first issue to consider from the perspective of public safety is how continually tethered dogs can pose a danger to humans [2,5,6,7]. The other side of the problem is that dogs are often tethered to protect the owner’s property. Such dogs are often tied for a long time, causing suffering or various behavioral problems [4,8,9]. Fatal dog attacks have increased in the USA (1979–2005) [10]. Gershman et al. [11] identified the tethering of dogs as one of the risk factors for increasing behavioral problems. However, the latter may also be due to the fact that during the time of Gershman’s study, both the number of dogs and the number of people owning dogs increased. [12]. In addition, the population of large dogs owned has increased by more than 45% [13]. Explanations for the increased number of attacks include less knowledge about animal behavior as well as the greater integration of dogs into the household [10].

The European Convention for the Protection of Pet Animals [14] promotes the welfare of pet animals and ensures minimum standards for their treatment and protection. The treaty was signed in 1987 and became effective in 1992 (on 1 May) after at least four countries had ratified it. Twenty-five countries have signed it, however, the Netherlands has not ratified the treaty yet.

Article 3 of the convention provides that nobody shall cause a pet animal unnecessary pain, suffering, or distress. In addition, Article 4 states that any person who keeps or looks after a pet shall provide accommodation, care, and attention that takes into account the ethological needs of the animal in accordance with its species and breed, in particular, taking all reasonable measures to prevent its escape. Dog chaining poses serious threats to a dog’s physical and psychological well-being. Due to the inhumane nature of continuous dog chaining, many cities and counties are passing local laws to ban the practice [15].

In recent years, the issue of keeping a dog under control by tethering has become controversial. Photos posted on social networks show emaciated dogs with chains ingrown into their neck skin, and permanently tethered dogs and animals without shelter, lacking feed and water. Such pictures provide evidence of the cruelty or ignorance of some people as well as potential violations of legislative provisions prohibiting animal cruelty [2].

In addition, protests and other social movements against the tethering of dogs call for legislative changes to ban or restrict tethering [8]. For example, individual legislative provisions determining the ways and rules of dog tethering have been adopted since 2020 in more than 20 states in the USA [16]. These provisions differ from state to state, but some elements are the same. In some states, tethering is allowed for a reasonable period, while in others, tethering is referred to as cruelty to animals. 

The authors of this article discuss the impact of dog tethering in terms of ethical shortcomings and behavioral problems as well as how these link to issues of public safety. The material in this article evaluates Slovak legislation, but also draws examples from other jurisdictions by way of comparison.

## 2. Animal Welfare and Behavioral Implications 

From an ethical point of view, “tethering is an unacceptable method of confinement for any animal and has no place in humane sheltering” [4]. “Constant tethering of dogs in lieu of a primary enclosure is not a humane practice, and the Animal Welfare Act U.S. Department of Agriculture prohibited its use in 1997 for all regulated entities” [17].

Animal welfare is described as physical and psychological harmony of an individual in relation to the environment in which the individual lives and actively spends its time. Welfare benefits for animals can be achieved by following minimal standards known as the “Five Freedoms” (the five animal welfare needs). One of these comprises the freedom to express normal behavioral patterns [18]. Behavioral manifestations of dogs as companion animals include the formation of social interactions either with individuals of their own species or with humans. If this need is not fulfilled, or the dog is restrained for an extended time, legitimate concerns arise about the well-being of the animal. The long-term tethering of a dog provides an example of one such restraint [9].

Tethering significantly limits the ability of a dog to satisfy its basic life needs. If the dog is tethered outside for most of its life, it faces the risk of physical damage, neglect, and health problems [19]. Examples of such problems includes the freezing of water supplied for drinking, attack by wild animals or other dogs, entanglement in chains, and defecation and sleeping in the same space [20,21].

Tethering limits the space available to the dog and increases the probability of dangerous defensive responses to perceived intrusions against resources (feeding bowl, water, territory) that the dog will instinctively protect [22]. Dogs are naturally territorial animals and when restricted to a small space in which they have to live for a long time, they perceive it as their safe shelter and may respond aggressively and territorially to the approach of people and manifest their emotions by barking or biting [23,24].

Animal Protection of New Mexico, a nongovernmental organization devoted to animal well-being, [25] commented that while chaining does not always make a dog aggressive, the animal is being given fewer options in fight-or-flight circumstances, thus inviting situations that increase the likelihood of aggressive responses. Chained aggressive dogs are time bombs and a risk to public health. There are many described cases where a chained dog or a dog that had broken loose from their chain has attacked and bitten a person, most frequently a child [26,27].

Dogs also need to become part of a social group. As they are social animals, it goes without saying that dogs not only need exercise, but also need regular interaction with other dogs and people [28]. In this respect, a number of studies [29,30,31,32], have already evaluated the influence of housing conditions on the behavior of different dog breeds. The results of these studies indicated that the dimensions of the allocated space and activity should be considered when evaluating the psychosocial well-being of dogs based on behavior, stress physiology, and injuries. Nevertheless, the dogs that were kept in pairs, or at least had a chance to see each other, spent more time sleeping, showing the tendency to spend less time vocalizing compared to the dogs kept alone. This indicates that social contact is important for better welfare.

When evaluating the welfare of dogs, social isolation can be as detrimental or even more adverse than spatial restriction [33]. Isolation of dogs and their limited contact with humans can result in excessive barking, running away, aggressiveness, or even apathy due to boredom and frustration [24,34,35]. Keeping the dog in isolation (for example, in one’s garden) may be considered as some form of unlawful conduct with elements of abuse as isolation from the family is a tough form of punishment for the animal [20].

Tethered dogs frequently exhibit additional problems [31,36,37,38]. In animals that have had to live in a severely impoverished or restricted environment, it is very likely that some form of stereotypical behavior develops [39,40]. Signs of behavioral problems that are indicative of stress and emotional distress include stereotypic (repetitive) pacing, spinning, running to and from, chewing, digging, and excessive self-licking, even to the point of self-mutilation [41,42,43]. Many such dogs bark and whine incessantly, which often creates tension in the neighborhood, increasing the risk of dogs being poisoned or physically injured.

Anxious and scared dogs that cannot escape from humans and animals may resort to lunging, tearing, or biting as a way of protecting themselves [44].

It is important to note that it is not possible to determine whether the problematic behavior is caused by tethering itself, or by isolation as such. To clarify this, future research should compare the responses of animals that are tethered and housed individually with animals that are under the same housing conditions, but not tethered.

## 3. Public Safety Implications (Risk for Humans)

As above-mentioned, dogs exposed to social or environmental restrictions show a tendency to become reactive, which is frequently the manifestation of their fear and subsequent aggressiveness. A study by Romaniuk, Flint, and Croney identified chaining as a risk factor for dog bites [9]. In this study, the authors were the first to use a multidimensional approach to determine dog-specific factors independently associated with biting. Several environmental factors were also associated with biting. Biting dogs were more likely to be chained in the yard and stay in houses with one or more children under 10 years of age. Of the 83 dogs chained in the yard, 53% growled or snapped at visitors to the house. 

On the other hand, a dog may be chained as a result of aggressive behavior, which may be a risk factor for biting a person. One study [26] estimated that 17% of reported dog bites and deaths nationwide between 1979 and 1998 were caused by dogs restrained (chaining included) on their owners’ property at the time of the attack. Although chaining is one of the types of restriction mentioned in this (and other) studies, the correct interpretation of these analyses may be complicated due to the circumstances associated with serious injuries and the extent to which they are reported correctly [45,46,47].

Another study [27] revealed more information about the risks of chaining dogs. The author identified incidents associated with severe dog bites. In eight of the 16 cases discussed, the dog involved was either chained or had broken loose from its chain to attack the victim.

Research on the correlation of bites [48] has focused on limited sets of variables and produced conflicting findings. The study correlates the dog bites by exploring a comprehensive set of variables related to the dog breed, the nature of its surroundings, and the circumstances of the bite. Based on multiple regression, the bites were mostly caused by a neighborhood dog that escaped from its home or yard, and the victim was most likely bitten in their own yard. The breed of dog was not correlated with bites in multiple regression. In most cases, it is the result of interaction between human behavior and the temperament of the dog that creates a risk, bringing together a combination of factors such as leaving the dog chained in the yard, other types of improper socialization, and the victim’s actions such as harassment or restraining the dog [49].

There are several inferences the reader can draw from these findings. First, it seems that dog bites are most likely the result from human error such as allowing dogs to roam the neighborhood and improper isolation in yards or homes. Dogs that are tethered in yards or otherwise kept outside often do not have proper socialization and can be a risk when escaping from the yard or tether [49,50,51].

## 4. Current Situation in Slovakia 

### 4.1. Chaining and Tethering of Dogs in Slovakia 

In order to better explain the situation involving dog tethering in Slovakia, we present the results of an unpublished questionnaire focused on the welfare of dogs in Slovakia in a variety of locations and regions. This survey was conducted between 2018–2019 by means of social networks and the distribution of personal questionnaires in the locations of socially disadvantaged families without access to the Internet. The participants among themselves kept 501 dogs. Preliminary results indicated that urban dogs were kept mostly in apartments (78.2%) and only some dog owners kept their animals free in their gardens with doghouses arranged for resting, or in cellars or other shelters. The situation was different in villages where 57.68% of owners kept their dogs free in their yards and only 16.48% shared apartments or houses with their animals. In rural areas, the dog was frequently kept in a pen with a doghouse (14.98%). This is also the way in which hunting and service dogs are frequently kept, with them being allowed into a run daily, or at least with having free access to the garden. 

A relatively high percentage of dogs were kept tethered next to houses in villages (10.86%) (Table 1). We were not able to determine whether they were tethered permanently or temporarily. However, we know that most of the dogs tethered in villages (36) were owned by very poor families. The owners stated that their dogs were kept free, however, most of the time the dogs were free, this occurred without supervision and frequently without any basic care.

### 4.2. Legislative Provisions Concerning the Keeping of Dogs in Slovakia

A fundamental principle of animal welfare states that animals must be considered as sentient beings. This principle should therefore underlie all EU legislation and policy that has any impact on living animals. At present, not all aspects are considered [52]. Animals are still the occasional recipients of deliberately inflicted abuse. Although generally the infliction of suffering is prohibited, an owner still commands total control over the destiny and purpose of the animal’s life [53]. 

The formerly adopted Animal Protection Act in Slovakia in 1995 [54] and relevant Executive Provision [55] sets the basis for the protection of animals by setting out rules of behavior of people to animals as well as details on the keeping of dogs. The provision regarding the keeping of dogs states that dogs kept outside can be long-time tethered using only a free sliding chain or cable attached to a trolley system that is at least three meters long and allows the dog to move freely for at least two meters on both sides. Other methods of tethering can be used only where necessary, for a maximum of eight hours, provided that the tethering device is at least 2.5 m long and equipped with two rotating pins preventing its shortening. Long-time tethering of a pregnant bitch in the last trimester of pregnancy (dogs are pregnant for three trimesters, each about 21 days long), a lactating bitch, a sick dog, or a puppy younger than six months is banned. The Act on Veterinary Care [56] also repealed both the Animal Protection Act [54] and Executive Provision [55].

Currently there is only one normative legal act governing the protection of animals in Slovakia. The Act on Veterinary Care [56] contains a broad definition of unlawful actions that amount to cruelty against animals. Article 22 deals with the protection of animals and apart from justified medical and approved experimental reasons, defines cruelty to include actions that, inter alia:

Cause permanent or long-term damage to the health of the animal, result in permanent or long-term disturbances of the animal’s behavior, exceed biological faculties of the animal or cause the animal undue pain, trauma, or suffering by using a stimulus, tool, or device that causes pain, clinically evident trauma, or clinically provable negative changes in the functioning of the nervous system or other organ systems of the animal. The animal is kept in unsuitable conditions or in a way that the animal itself is involved in the activities that cause it pain and suffering or the animals mutually cause themselves pain and suffering.

These provisions also involve the protection of animals with respect to their suffering caused by long-term or permanent tethering. The failure to exclude stressors of various character, whether biological, physical, or chemical, can also result in cruelty. The physical stress factors include the use of an unsuitable muzzle that limits the dog’s breathing, a collar with spikes, or unsuitable constricting collar or tethering (not only) by chains; all of this is considered a violation of the Act.

The recently adopted Decree of the Ministry of Agriculture and Rural Development of Slovak Republic [57] set the details of the protection of companion animals, requirements on quarantine stations and animal shelters, also deals with the keeping of dogs and includes some parts of the Executive Provision [55] above-mentioned. 

General requirements on the protection of companion animals include rules that determine the obligations of the owners (keepers) of these animals. The rules state that the owners/keepers are obliged to ensure daily inspection of their animals, particularly with regard to the animals’ behavior, body condition, and nutritional status, and owners/keepers have to ensure their animals have sufficient exercise according to the species. Moreover, in the case of dogs, the amount of exercise should take into account the size and temperament. 

The Decree also provides more specific requirements for the protection of dogs as follows: “The collar of tethered (chained) dog must be sufficiently wide, with adjustable perimeter, and the weight of chain must be adjusted to the size and weight of the dog in order to prevent difficult breathing and cutting into his neck or damaging his health. If the chain can move over a guiding cable, the cable shall be long enough to allow the dog lengthwise movement for at least 5 m and enough space shall be provided for 2 m movement on both sides. If the chain is attached any other way, its length shall be at least 4-fold of the body length of the dog measured from the tip of his nose to the root of his tail, but not shorter than 2.5 m, and must be equipped with two rotating pins to prevent its shortening due to its tangling up, and arranged in a way so it cannot become entangled to any object in the run. The attachment of the chain shall allow the dog to move over the area shown in Table 2 and hide in an easily available shelter against adverse weather, particularly against rain, frost, and direct sunlight.”

### 4.3. Animal Law Enforcement in Slovakia

Pet animals are susceptible to many forms of animal abuse at the hands of their owners or keepers including restraining dogs with the aim of preventing their escape. 

In addition to situations already discussed concerning failure to ensure the well-being of animals resulting in detrimental effects on canine behavior, there were also extreme examples such as people hanging animals. These situations were caused by targeted activities of the abuser. There were also unintentional or incidental situations when the animal was restrained to a stationary object (mostly chained dogs) and the hanging was caused by the animal’s own movements. 

Other cases may be related to the inattentiveness of the owner or keeper who is responsible for the animal and the failure of the owner or keeper to take appropriate action, for example, not ensuring necessary veterinary treatment or medication that the animal needs (e.g., after injury) [58]. As a rule, such abuse or neglect resulting in death occurs together with the exposure of the animal to adverse conditions (e.g., keeping the dog outside in a pen in extreme heat or cold, restrained by a short chain wrapped round the neck, frequently ingrown into live neck tissue, in toxic environment, etc.). According to Munro and Munro [59] and Rogers and Stern [60], asphyxiation in animals may occur under a variety of circumstances ranging from non-accidental injury in the form of strangulation (ligature strangulation, hanging) through suffocation. Animals may be strangled intentionally by an attack or as a result of self-inflicted damage (a dog pulling incessantly on a collar or choke-chain) [61]. Positional asphyxia is a type of asphyxia where the position of an animal compromises the ability to breathe.

Enforcing compliance with relevant regulation regarding tethering of dogs by animal protection inspectors is met with difficulties because they cannot properly monitor the needs of animals according to the tethering only for a necessary period of time. Due to this, the Freedom for Animals Association launched a petition to ban the chaining of dogs in Slovakia. According to this association, dog owners may use other ways of restraining and this would prevent the necessity to amend the legislation. It is imperative that society complies with the rules set by the Act on Veterinary Care [56] and regulators step up inspections. In addition, the Civil Law [62] recognizes live animals as sentient beings and in this way, assigns them a special status because they are valued for their capability of perceiving by their own senses. Additionally, rules applying to movable assets apply to animals, but there are exceptions to these rules when they contradict the concept of a live animal as a sentient living being. Animals are treated as property in many jurisdictions around the world, but they are subject to anti-cruelty laws and/or other welfare provisions. This means that as sentient beings, they are protected by law. On the other hand, they can be, for example, sold, or donated, yet this cannot happen to other “sentient beings” such as humans. 

It is important to concentrate on the specifics of keeping conditions with regard to the species and individual characteristics of the different animal. Obviously, this involves the provision of feed and water and how it is ensured, the availability of a run, hygiene and cleanliness of the housing spaces, avoidance of objects and structures that present risks to the life and health of animals (e.g., sharp edges), and the availability of shelter in case of adverse weather conditions (pen or doghouse providing protection against, sun, rain, snow, frost, draft, etc.).

In the case of animal abuse, it is important to inspect the animal and document the situation (position and visual observations, e.g., potential skin defects, hair cover; particularly its overall health, nutritional state, and body condition). The immediate result of a veterinary check-up represents the official record of material observations at the time of inspection. This record then details any observable violations of anticruelty regulations as well as the failure to meet positive obligations pursuant to the Veterinary Regulations. This official record may also contain restorative orders to deal with the neglect and failure of positive obligations.

To mention one example, a veterinary inspector reported the following offence committed in Slovakia that involved cruelty to animals: the owner kept his dog behind his house restrained by a 40 cm long chain at temperatures well below zero (−18 to −20 °C, even below that for longer periods) without food or the possibility of movement, considerably undernourished and in poor health. The cruelty to this dog involved hunger and overall neglect. Due to the offense, the owner was punished with a fine. 

In an analogous case, police information revealed an owner who kept two cattle and one dog on land behind the family home. The animals were all kept on short chains and the lack of exercise, shelter, food, and water resulted in the death of those animals. Consequently, the owner was charged with abusing the animals to death. Contrary to the previous case, three animals died and this could qualify as malpractice in terms of the Slovak penal code and the owner was punished with imprisonment.

## 5. Discussion and Future Directions

The issue of dog tethering is complicated and publications about the tethering of these animals present controversial information. Proposers of laws against the tethering of dogs frequently refer to statistical data that indicate tethered dogs exhibit higher rates of aggression. However, the conclusions of studies on the harmful effects of tethering do not explicitly refer to tethering as the direct cause of such behavioral problems. It is impossible to determine unambiguously if the misbehavior of the dog was caused by tethering or previous or simultaneous neglect, abuse, provocation, or another factor [47,63]. 

Despite the amendment of Decree No. 283/2020 Coll. [57], not much has changed in practice as dogs can be restrained for various reasons, particularly if the protection of other animals or objects are of concern. As the relevant provisions state that dogs can be restrained for a necessary amount of time, a definition set out in the decree is highly subjective, particularly if the protection of life or property is concerned. The definition set out in the legislation (Decree No. 283/2020 Coll.) is as follows: “The dog may remain in a chain or other means of restraint (hereinafter referred to as ‘chain’) only for the time necessary to ensure the protection of human life or health, animal life or health, property or to ensure its well-being, in particular during feeding, cleaning of the breeding grounds, examination or treatment.” It is impossible to determine when and for how long the animals are tethered or released and inspectors have to rely solely on the statements of the owners, who usually aim to justify their practice. Chaining of dogs can be perceived as problematic by responsible owners who use it for the improvement of the animal’s life by protecting them against straying, escape, or performing some activities. Reasonable restraining of the dog with the availability of suitable shelter, adequate exercise, and fulfilling their basic needs may keep the animal safe, for example, until a fence between the neighboring lots is finished.

Starinsky [64] has researched the relationship between tethering dogs and escape rates. More specifically, his study aimed to determine the escape rates for dogs who were in some way restrained and to determine whether a history of biting was associated with the method of containment. His survey, which was conducted in Columbus, Ohio, investigated dogs confined to their owner’s property by a physical fence (78.0%) in most cases, an electronic fence (14.2%), or a tether system (7.8%). Dogs confined by an electronic fence escaped more frequently (44.0%) than dogs kept captive by a transparent fence (23.3%), a privacy fence (23.3%), or tether (26.8%). They also reported that 4.6% of dogs had reportedly bitten a person in the past and 7.7% another dog, but the containment method was not significantly associated with whether the dogs had ever bitten a human or another dog. The results suggested that the rate of escape, but not the history of biting, was associated with the method used by owners to restrict the dogs. 

The owners of sled dogs also tie them to doghouses that protect these animals against adverse weather. However, some argue that the advantages of tethering outweigh the possible disadvantages. Many mushers support tethering. Mushers use tethering for many reasons including economic benefits, freedom provided to dogs to interact with the environment and handlers [65], functionality on the trail, and prevention of dog fights [66]. Tying sled dogs allows individual care for each dog. Tethering also offers an easy way to track dogs and a housing system from which dogs cannot escape. Ties may have become popular with some mushers because it was once believed that tethered dogs had a higher level of activity and were therefore faster. However, scientific evidence now suggests that tethering does not have this benefit [67].

A group of scientists [66] compared the behavior of sled dogs that were chained with those that were kept in cages and did not observe differences in behavior in relation to their restraining. It was important that both groups, independent of their housing, had regular and adequate exercise. Given the different views on tethering, it is important to evaluate how tethering affects the overall physiological and behavioral welfare of the dog. This is particularly important in order to evaluate under what conditions, and how, the dog could be tethered. The way of tethering depends on the level of physical restriction for the intended purpose. Obviously, one universal procedure should not be applied to every breed or working class of dogs. Not many studies have examined the impacts of tethering and pen housing on dog behavior and welfare [68].

In order to draw a conclusion about the consequences of dog tethering for dog welfare, further research is needed that takes into account the different breeds and working classes of dogs.

Nevertheless, at this stage, it can be concluded that at the very least, correct placement of the dog’s collar is important as are other criteria including the unsuitability of long-term confinement and the absence of suitable shelter in extreme weather [13]. While these criteria lead to conditions antithetical to good animal welfare, the most important factor is the vigilance of the owner and the prevention of neglect. Dogs must be properly socialized, ideally kept in the home, spend time outside in a safe yard, and walked by the owners on a leash [49,69]. The dog, as a social animal, requires socialization, the owner’s company, and enough movement to be able to perform natural behavior [70]. Therefore, future research should focus on the impact of the long-term tethering of dogs on the possibility to manifest a natural behavior. In addition to behavioral indicators, it is also necessary to focus on the evaluation of physiological parameters in order to be able to assess the effect of tethering on welfare.

### Do We Have Alternatives to Tethering and Chaining? 

Humane tethering alternatives include fenced yards with a doghouse or another covered area, large pens, cable/trolley runs, invisible/electric fences, all with access to water and food, and with weather protection. Installing a secure fence for a dog, of an appropriate height, gives a dog unlimited freedom. Another option is installing a dog run. A dog run is an affordable alternative to building a fence and still allows the pet the opportunity to stretch its legs. Some dogs often run away from their owners. To prevent animals from climbing over a fence, a Coyote Roller (roller designed to prevent a dog from climbing over a fence) or a jump restraint harness can be used. Sterilization of the dog can also solve the problem. A neutered male dog is less likely to try to escape a fence or mark inside the house. A spayed female dog will not roam around looking for a mate [71]. Every dog should receive adequate behavioral training. Teaching people that dogs are social beings that should not be isolated on chains is very important. Dogs are part of the family. Of course, it is necessary that they have regular exercise and are provided with adequate attention, food, water, and veterinary care.

Regardless of what tethering alternatives are used, experts agree that dogs should be allowed to socialize with their owner/keeper and household members.

## 6. Conclusions

There are many arguments that both support and oppose dog tethering. We are convinced that in any method of confinement, it is necessary to take into account the preference of the dog, and only then its effectiveness. Therefore, further study is needed for a thorough conclusion and a better understanding of the impact of tethering on the dog’s welfare.

The question is whether the ban on chaining is the best solution. There is a low probability that this ban will change the behavior of violators of legislative provisions who are cruel to dogs. Poorly thought legislative amendments may adversely affect those who observe current regulation and restrain their dogs in a safe and humane manner. Part of the problem stems from the way animal protection inspectors control and enforce the regulation with respect to tethering. In this regard, those who enforce the law need to focus on specific conditions that respect the needs of different breeds of dogs as well as individual dog preferences and characteristics. The most important thing, however, is to raise people’s awareness, especially through campaigns where they obtain information on the potential consequences to animal health and animal suffering, which can be caused by long-term confinement of dogs.

## Figures and Tables

**Table 1:** Ways of housing of dogs in Slovakia.

Type of Settlement	Number (N)	Free in a Yard (%)	Tethered (%)	Pen with a Doghouse (%)	In a House or Apartment (%)
Town	234	20.52	0.43	0.85	78.20
Village	267	57.68	10.86	14.98	16.48

**Table 2:** Minimum requirements on free area of the pen (Regulation No. 283/2020 Coll., Supplement 3).

Number of Dogs	Mean Weight of Dog Free Pen Area (m^2^)					
	Up to 5 kg	5–10 kg	10–20 kg	20–30 kg	30–50 kg	50 kg or More
1	3.5	4	4.5	5	6	7
2	3.5	4.5	5	6	8	9
3	4	5	5.5	7	10	11
4	4.5	5	6.5	9	12	13
5	4.5	6	7.5	11	14	15
6	5	7	9	13	16	17
Each additional dog	+0.5	+1	+1.5	+2	+2	+2.5 ^1^

^1^ It is not recommended to keep more than six dogs of this weight category in one common pen.
